# Analysis of Flavor Differences in Yak Milk Powder at Different Milk Production Stages by Headspace Solid-Phase Microextraction and Gas Chromatography–Mass Spectrometry

**DOI:** 10.3390/foods14010091

**Published:** 2025-01-01

**Authors:** Diandian Wang, Yaxi Zhou, Jian Zhao, Yu Guo, Wenjie Yan

**Affiliations:** 1 College of Biochemical Engineering, Beijing Union University, Beijing 100023, China; spwdd2018@163.com; 2 Beijing Key Laboratory of Bioactive Substances and Functional Food, Beijing Union University, Beijing 100023, China; 18810747451@163.com (J.Z.); gyanling9408@sohu.com (Y.G.); 3 Institute of Apicultural Research, Chinese Academy of Agricultural Sciences, Beijing 100093, China; 15239407080@163.com

**Keywords:** yak milk, volatile compounds, HS-SPME-GC-MS, OPLS-DA, hierarchical clustering

## Abstract

The aroma of yak milk powder is a crucial sensory indicator for evaluating its quality and flavor. Yak milk powders collected from different lactation periods exhibit distinct flavors, but no studies have thoroughly investigated the aroma characteristics and variation patterns of yak milk powders across these periods. This study identified and analyzed the volatile compounds in freeze-dried colostrum powder (YCSP), freeze-dried mature milk powder (YMMP), and freeze-dried ending milk powder (YEMP) using headspace solid-phase microextraction combined with gas chromatography–mass spectrometry (HS-SPME-GC-MS) and multivariate statistical analysis. A total of 48 volatile compounds were identified, with significant differences in the types and contents of these compounds across the three samples. Compared to YCSP and YEMP, YMMP contained higher levels of acids and esters, while the levels of alkanes and alcohols were lower. Principal component analysis (PCA), orthogonal partial least squares discriminant analysis (OPLS-DA), and hierarchical clustering heatmap analysis revealed a high degree of differentiation and notable variation in volatile compounds between the samples from different lactation periods. Key compounds such as aldehydes, alcohols, and esters were found to distinguish the lactation stages, with certain compounds more prevalent in colostrum and others in mature and ending milk. These findings suggest that the methodologies employed—HS-SPME-GC-MS combined with multivariate analysis—can effectively distinguish flavor differences among yak milk powders from different lactation periods. This approach allows for the rapid and comprehensive analysis of volatile components in milk powders, aiding in the identification of collection periods and providing valuable insights for improving the flavor quality of dairy products. Furthermore, the results can benefit the dairy industry by enhancing product development, quality control, and flavor profiling of milk-based products across different stages of lactation.

## 1. Introduction

Yaks are primarily distributed in the pristine alpine pastures around the Qinghai–Tibet Plateau, where their food sources consist of natural, pollution-free grasses [1]. Consequently, yak milk and yak milk powder, as natural green foods, are highly favored by consumers, especially the pastoralists in high-altitude areas [2,3]. Yak milk powder is rich in various nutrients, including high levels of protein, milk fat, and numerous vitamins [4,5]. Therefore, yak milk powder serves as an important source of nutrition for the residents. In fact, yak milk and its dairy products are the only natural milk source in the Qinghai–Tibet Plateau region [5]. Yak farming plays a critical role in the economy of mountainous regions. In high-altitude areas where agriculture is limited, yak farming is a primary source of income, sustenance, and food security for pastoral communities. Furthermore, yak milk holds significant cultural value. It is not only a dietary staple but also an essential part of local customs, rituals, and festivals, serving as a symbol of tradition and community identity.

Research has found that the nutritional components of yak milk are not static. During the main lactation periods of yaks, the primary substances present in yak milk are protein, fat, and lactose [6]. However, the content of these key nutritional components can vary due to multiple factors, such as growth environment, seasonal changes, number of pregnancies, yak breed, and lactation stage [7]. For example, a study found significant differences in the volatile compounds of yak milk from three different regions in Gannan [8]. Studies have shown that the solid content in yak colostrum is higher, with protein levels approximately three times greater than in other lactation periods and fat content being two to three times higher than in other periods. Therefore, the composition and nutritional content of yak milk powder collected from different lactation periods also differ [9,10]. Compared to cow, goat, or buffalo milk, yak milk is particularly rich in fat and protein, making it more calorically dense and nutritionally beneficial for people living in high-altitude areas, where energy demands are higher [7,11].

Volatile compounds are key factors influencing the flavor and aroma of dairy products, playing a crucial role in the sensory characteristics, quality, and consumer acceptance of the products [12,13]. For instance, compounds such as esters, aldehydes, and alcohols are often associated with pleasant fruity and sweet aromas, which can enhance the overall taste experience of dairy products. In contrast, the presence of off-flavors, which may result from compounds like short-chain fatty acids, can lead to negative consumer perceptions. These flavor characteristics not only affect the quality of dairy products but also influence consumers’ purchasing decisions, as products with a favorable and distinctive flavor profile are more likely to be preferred [14]. Moreover, changes in volatile compounds are closely related to the quality stability and freshness of dairy products [15]. For instance, yak milk, with its unique growing environment and rearing practices, exhibits different volatile compound characteristics compared to common dairy products such as cow and goat milk. Previous studies have shown that cow milk contains higher concentrations of fatty acids and esters, while goat milk is characterized by a higher content of alcohols, which imparts a unique flavor. In contrast to these dairy sources, yak milk demonstrates its own distinctive volatile compound profile, offering a new perspective for flavor research [16]. Therefore, analyzing the volatile compounds in yak milk not only contributes to enhancing the quality of yak milk products but also provides new directions for dairy processing technologies and quality control.

Headspace solid-phase microextraction combined with gas chromatography–mass spectrometry (HS-SPME-GC-MS) has high sensitivity in detecting volatile compounds, making it commonly used for analyzing volatile compounds in food [17,18], especially in milk and dairy products [19,20]. Previous studies have confirmed that the differences in the types and contents of nutrients in yak milk powder can affect the variety and concentration of volatile compounds [8]. For example, certain fatty acids and esters, which contribute to the desirable fruity or creamy flavors in milk, are closely associated with higher fat content. Conversely, some undesirable volatile compounds, such as aldehydes, may be formed as breakdown products of lipid oxidation or protein degradation. Moreover, the correlation between nutrient content and volatile compounds in yak milk may vary depending on the lactation period, with fat content typically being the most significant factor in determining the overall volatile profile. However, there has been limited research on the volatile compound profiles of yak milk powder from different lactation periods.

Research on goat milk has shown that volatile fatty acids change during lactation, directly influencing the milk’s flavor characteristics [21]. Similarly, studies on donkey milk have revealed lactation-related variations in amino acids, which are important components contributing to the milk’s characteristic odor and flavor [22]. These findings suggest that the volatile compound profiles in dairy products can serve as indicators of lactation stages. Thus, we hypothesized that volatile compounds in yak milk powder made from milk collected during different lactation periods might exhibit certain differences, potentially allowing for the identification and classification of yak milk’s collection period by analyzing the volatile compounds.

In this study, we determined the volatile compounds in freeze-dried colostrum powder (YCSP), freeze-dried mature milk powder (YMMP), and freeze-dried ending milk powder (YEMP) using HS-SPME-GC-MS. Furthermore, we effectively distinguished the flavor differences among yak milk powder samples harvested during different lactation periods through Principal Component Analysis, OPLS-DA model analysis, and hierarchical clustering analysis. The research results provide valuable insights for a deeper evaluation of yak milk powder flavor and the identification of yak milk harvesting periods, which can benefit the dairy industry by enhancing product differentiation and quality control. Additionally, the methodologies used in this study can be replicated in other dairy product research to explore similar flavor profiles and volatile compound differences across different milk types and stages.

## 2. Materials and Methods

### 2.1. Collection of Yak Milk Samples

YCSP, YMMP, and YEMP were all provided by Zangyuan Zhaomei Dairy Products Co., Ltd. (Seda County, Ganzi Tibetan Autonomous Prefecture, Sichuan Province, China). The collection dates for YCSP, YMMP, and YEMP were April, June, and October of 2023, respectively. Milk from six yaks was collected for each lactation period and then mixed in equal proportions to form a single experimental sample. The age of these yaks ranges from 5 to 7 years, their diet consists of alpine herbaceous plants, and their health status was ensured to be good to guarantee the representativeness of the samples. The geographic location of these yaks is in the Qinghai–Tibet Plateau region, where they graze on natural, pollution-free alpine grasses. This unique environment, characterized by high altitude and cold weather, can influence the composition of yak milk, particularly affecting its protein, fat, and mineral content due to the nutritional profile of the local forage and climatic factors. The collected yak milk was pasteurized at 72–80 °C for 15 s. It was then freeze-dried using a freeze dryer (FD503, Jinan Junde Instrument Co., Jinan, China). The resulting YCSP, YMMP, and YEMP were subsequently collected and stored at −20 °C until analysis. Samples were transported under frozen conditions to maintain stability during transit.

### 2.2. Reagents

Ethyl decanoate was purchased from Shanghai Pharmaceuticals Holding Co., Ltd. (Shanghai, China), with a purity of ≥99.5%. The n-alkane C7–C40 standard mixture was obtained from Sigma-Aldrich Supelco (Shanghai, China).

### 2.3. Headspace Solid-Phase Microextraction (HS-SPME)

Volatile compounds in different yak milk samples were separated using a modified method based on previous studies [23]. The samples were extracted using an automated headspace sampling system equipped with a 50/30 µm DVB/CAR/PDM extraction fiber (Supelco, Bellefonte, PA, USA) and an MPS2 XL multifunctional autosampler (Gerstel, Mülheim an der Ruhr, Germany). A 1.0 g sample was accurately weighed and placed into a 20 mL headspace extraction vial, with a suitable internal standard solution (5 µg/L ethyl decanoate) added. The vial was then incubated with shaking at 70 °C for 30 min. Following this, the extraction fiber was immediately inserted into the headspace vial, and extraction was carried out for 30 min. The volatile compounds were then desorbed in the GC-MS injector (250 °C) for 5 min [24].

### 2.4. Gas Chromatography–Mass Spectrometry (GC-MS) Analysis

Based on previous methods with appropriate modifications [25], GC-MS analysis was performed using an Agilent 7890A-5975C system (Agilent, Santa Clara, CA, USA). The chromatographic separation was conducted on a DB-WAXETR capillary column (30 m × 0.32 mm × 0.25 µm, Agilent, Santa Clara, CA, USA). The injection port temperature was set to 250 °C, and the injection was performed in spitless mode. The chromatographic program was as follows: the initial temperature was held at 40 °C for 5 min, then increased to 250 °C at a rate of 5 °C/min, and held for 10 min. Helium was used as the carrier gas with a flow rate of 1.7 mL/min. The MS system operating conditions were as follows: the ion source temperature was set to 230 °C, the quadrupole temperature was set to 150 °C, and the interface temperature was set to 280 °C. The electron ionization (EI) energy was set to 70 eV, with a scanning range of 35–500 *m*/*z*.

### 2.5. Statistical Analysis

Volatile compounds were identified by searching the National Institute of Standards and Technology (NIST) mass spectral database for compounds with a similarity score > 80% and by calculating the retention index (RI) of the volatile compounds using an n-alkane C7–C40 standard mixture. The RI values were then compared with those in the database to identify the compounds [26]. OPLS-DA plots were generated using the OmicStudio tools available at https://www.omicstudio.cn/tool (accessed on 15 October 2024). Additionally, a hierarchical clustering heatmap was created using the heatmap tool available at https://www.omicstudio.cn/tool/107 (accessed on 15 October 2024) to visually compare and analyze differences in volatile compounds among samples. All experiments were conducted in triplicate, and the results are presented as mean ± standard deviation (SD). Statistical analysis was performed using one-way analysis of variance (ANOVA), followed by Duncan’s post-hoc test. A *p*-value of <0.05 was considered statistically significant.

## 3. Results and Discussion

### 3.1. Identification and Quantitative Analysis of Volatile Compounds in YCSP, YMMP, and YEMP

**Figure 1:**
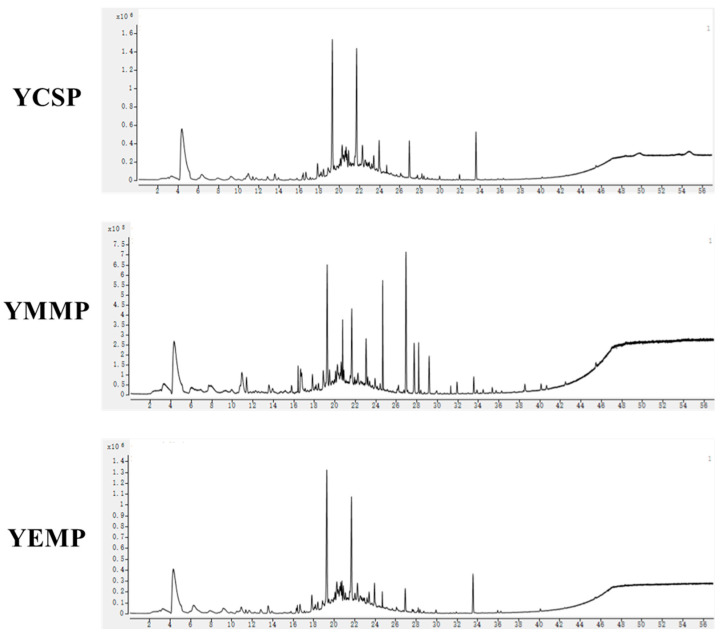
Total ion chromatograms (TIC) of YCSP, YMMP, and YEMP samples. The X-axis represents the collection time (min), and the Y-axis represents the signal intensity.

The nutritional content of yak milk powder obtained by freeze-drying yak milk collected during different lactation periods varied, and the differences in nutritional components led to distinct flavors and sensory characteristics of the yak milk powder. The characteristics of volatile compounds in yak milk could also reflect its quality and determine the degree of consumer acceptance and preference [13]. To compare and analyze the differences in volatile compounds among yak milk powders collected at different lactation periods, we used HS-SPME-GC-MS to analyze the volatile compounds in YCSP, YMMP, and YEMP samples. The total ion chromatograms of the three yak milk powder samples are shown in Figure 1.

Table 1 shows the information on the volatile compounds identified in the YCSP, YMMP, and YEMP samples. A total of 48 volatile compounds were identified across the three yak milk powder samples, and the content of each volatile compound varied significantly among the samples. All three samples contained higher levels of pentadecane, hexadecane, and dimethyl ether. Notably, the YCSP milk powder sample contained large amounts of 6,9-dimethyl-tetradecane, 2-phenoxy-ethanol, and heptadecane. The YMMP milk powder sample had higher levels of butyric acid and caproic acid, while the YEMP milk powder sample contained more toluene and 3-ethoxy-3-methyl-2-butanone. Previous studies analyzed the flavor changes in yak milk under different storage temperatures and times using HS-SPME-GC-MS and identified 23 volatile compounds (retention match score above 600) from yak milk [13]. Additionally, our previous research used headspace gas chromatography–ion mobility spectrometry (HS-GC-IMS) to determine the changes in volatile organic compounds in yak milk powder under different drying methods, identifying 17 volatile compounds [27]. Similarly, we detected volatile organic compounds in yak milk powder under different sterilization conditions using HS-GC-IMS and identified only 17 volatile compounds [28]. Compared to the HS-GC-IMS method, the majority of the compounds identified in this study were identified for the first time. Clearly, our current study identified a greater variety of volatile compounds, which may be attributed to the more comprehensive compound coverage in the database we used.

### 3.2. Comparative Analysis of Volatile Compounds in YCSP, YMMP, and YEMP Samples

**Figure 2:**
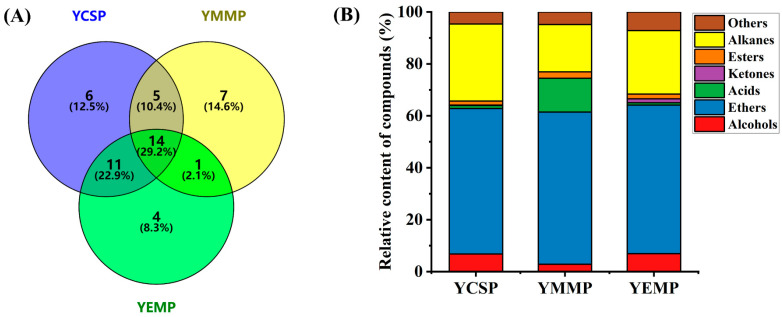
Venn diagram of identified compounds in YCSP, YMMP, and YEMP samples (**A**) and stacked bar chart of the relative content of various compound types (**B**).

We further classified and statistically analyzed the identified volatile compounds. The results are shown in Figure 2. Venn diagrams can reflect the distribution relationships between different sample data and are often used in the field of food flavor research to compare the distribution of volatile compounds among different samples [23,29]. According to the Venn diagram results, the numbers of volatile compounds detected in the YCSP, YMMP, and YEMP samples were 36, 30, and 27, respectively. The unique volatile compounds in the YCSP, YMMP, and YEMP samples were six, seven, and four, respectively, while 14 volatile compounds were shared among the three yak milk powder samples. Additionally, excluding the volatile compounds shared by all three samples, 11 volatile compounds were common between the YCSP and YEMP samples, five were shared between the YCSP and YMMP samples, and only one volatile compound was shared between the YEMP and YMMP samples (Figure 2A). This indicated that the types of volatile compounds in the YCSP and YEMP samples were more similar, while there was a greater difference in the types of volatile compounds between these two samples and the YMMP sample.

The 48 volatile compounds identified from the three yak milk powder samples were mainly classified into six categories: alcohols, ethers, acids, ketones, esters, and alkanes. As shown in Figure 2B, the main volatile compounds in the three yak milk powder samples were ethers and alkanes. Additionally, it was evident that the types and contents of volatile compounds in the YCSP and YEMP samples were quite similar, while the YMMP sample contained higher levels of acids and esters and lower levels of alkanes and alcohols. This result corroborated the findings from the Venn diagram, suggesting that the compound composition in the YCSP and YEMP samples was more similar, whereas the YMMP sample had a markedly different composition.

The variations in volatile compounds in yak milk from different lactation periods have significant biochemical implications. For instance, compared to YCSP, YMMP contains higher concentrations of esters and aldehydes, which may reflect changes in the fat metabolism process of the milk or be linked to microbial activity and enzyme expression. These changes directly influence the flavor and nutritional composition of the milk. Additionally, the changes in volatile compounds are closely related to the nutritional components of yak milk. For example, the increase in aldehydes and alcohols in YMMP may be associated with higher fat content, as these compounds are typically generated during the fat breakdown process.

**Figure 3:**
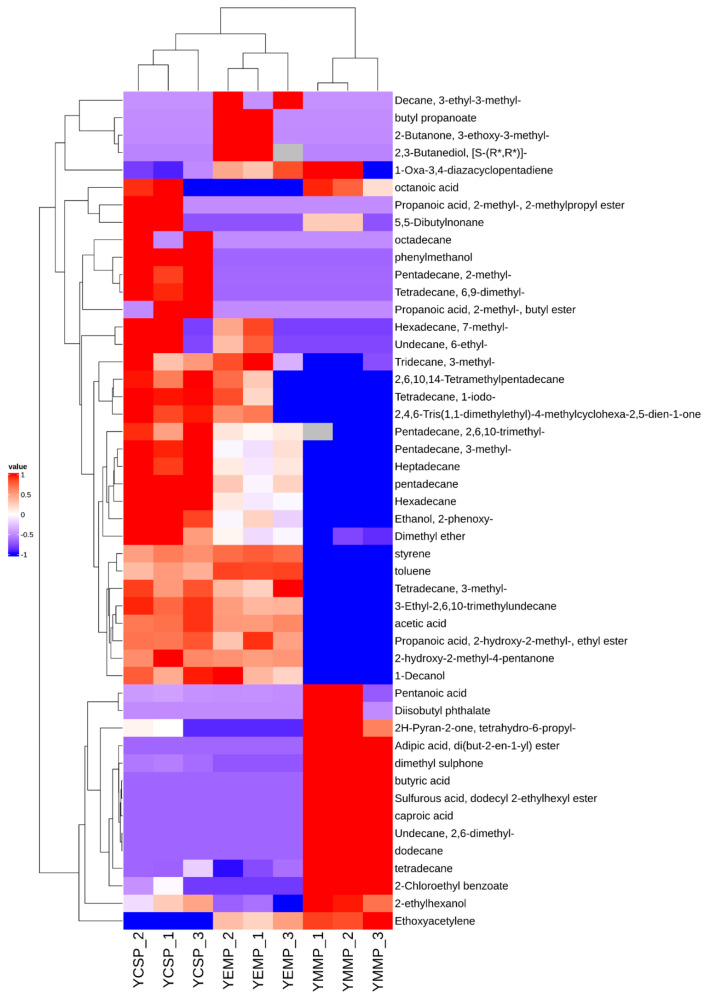
Hierarchical clustering heatmap of volatile compounds in YCSP, YMMP, and YEMP samples. The horizontal axis represents the sample names, and the vertical axis represents the detected volatile compounds. Blue squares indicate relatively low levels of the compounds in the samples, while red squares represent relatively high levels.

Hierarchical clustering is a commonly used method in clustering analysis that replaces the relationships between individual samples with the distances between them, gradually clustering samples that are close in distance into a group step-by-step to complete the clustering process [30,31]. Combining hierarchical clustering with heatmaps allows for a clear visualization of the data distribution between sample datasets [32]. Therefore, we further conducted hierarchical clustering heatmap analysis of the 48 volatile compounds identified from the three yak milk powder samples, and the results are shown in Figure 3.

The results in Figure 3 indicated that the relative contents of volatile compounds varied among the different yak milk powder samples, with the distributions of volatile compounds in the YCSP and YEMP samples being more similar, while those in the YMMP samples were mainly located in the lower right corner. The row clustering process followed a bottom-up approach, and as clustering progressed, YCSP, YMMP, and YEMP each formed individual clusters. As clustering continued, YCSP and YEMP samples gradually clustered together, while YMMP did not join this cluster. This further demonstrated that there were differences in the volatile compounds among the YCSP, YMMP, and YEMP samples, with smaller differences between YCSP and YEMP and larger differences between these two and YMMP.

### 3.3. Principal Component Analysis

**Figure 4:**
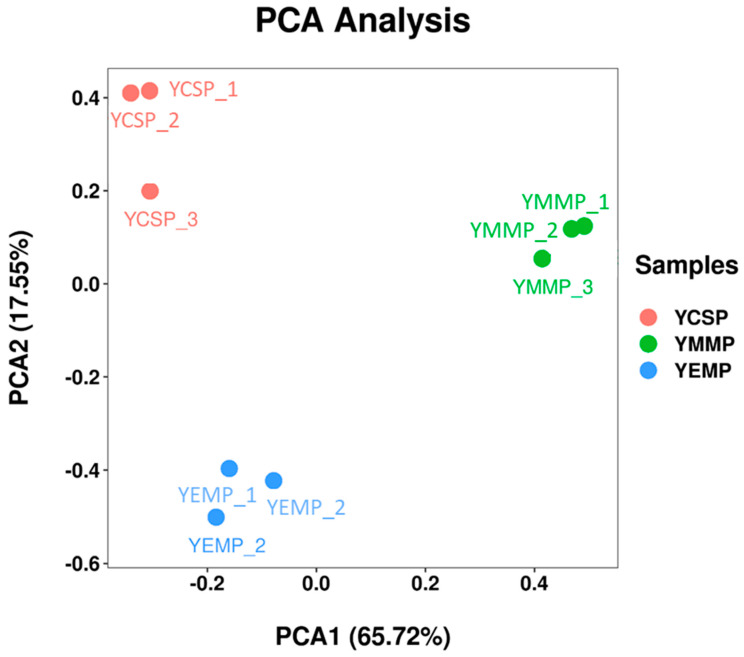
Principal Component Analysis (PCA) of volatile flavor compounds in yak milk across different lactation periods.

Principal Component Analysis (PCA), as a multivariate statistical method, can reduce the dimensionality of data to eliminate unnecessary information, thereby examining the correlation between different variables [27,33]. To further analyze the differences among the yak milk powder samples, we conducted PCA analysis using the content data of the 48 identified compounds, and the results are shown in Figure 4. Similar previous studies suggested that, when the cumulative contribution rate of Principal Component 1 (PC1) and Principal Component 2 (PC2) is greater than 80%, the PCA model can be considered relatively reliable as a separation model [30,34].

As seen in Figure 4, the contribution rate of PC1 was 65.72%, and that of PC2 was 17.55%, with a cumulative contribution rate of 83.27% for PC1 and PC2. Therefore, the PCA model was considered reliable. In the PCA plot, the higher the clustering degree of sample points, or the closer the distances, the smaller the differences between the samples. Conversely, greater distances indicate larger differences. Thus, the PCA plot can reflect the variability between samples from a global perspective [35]. In Figure 4, the data points for YCSP, YMMP, and YEMP showed high clustering within each group, while the data points among the three groups were far apart, indicating significant distinctions. In conclusion, there were clear differences in volatile compounds among YCSP, YMMP, and YEMP, and PCA analysis could be used to visually differentiate the volatile compounds in yak milk powders made from the milk collected during different lactation periods.

### 3.4. OPLS-DA Model Analysis

**Figure 5:**
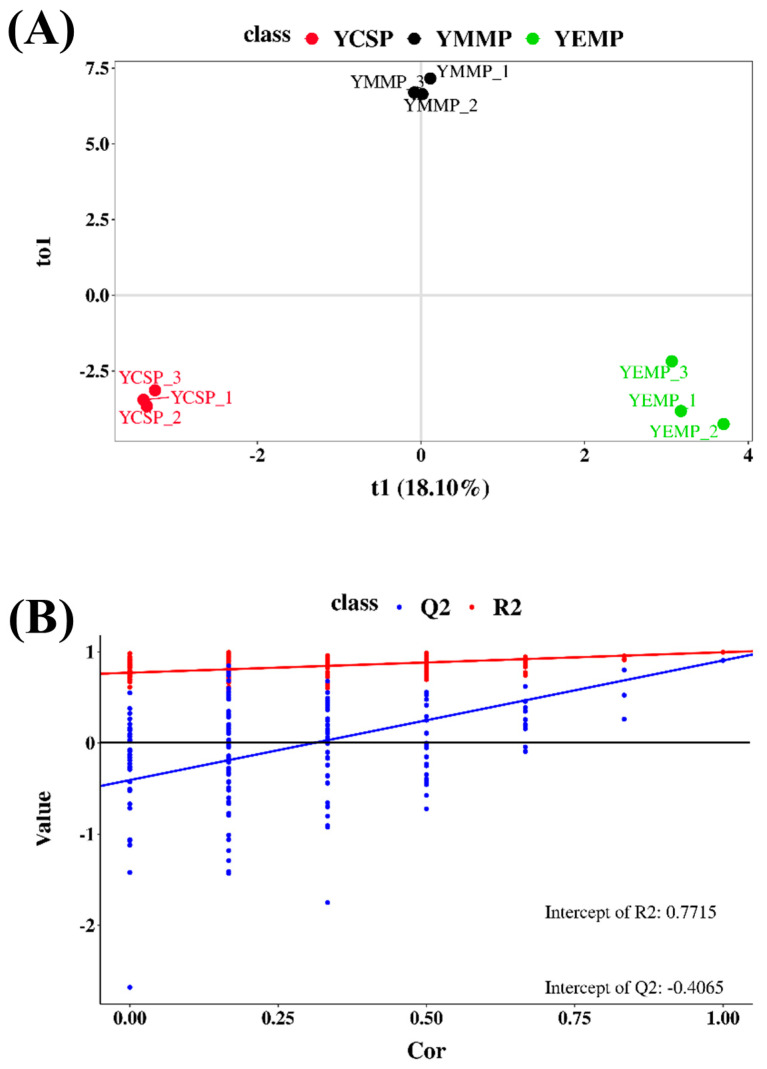
OPLS-DA model analysis results. (**A**): OPLS-DA score plot. (**B**): OPLS-DA model validation plot (results of 200 permutation tests).

The Orthogonal Partial Least Squares Discriminant Analysis (OPLS-DA) model, as another multivariate statistical method, was very helpful for the visualization of high-dimensional data [36]. To explore the relationship between volatile compounds and the groups of yak milk powder samples, we further established an OPLS-DA model to perform discriminant analysis on the volatile compounds in yak milk powders from different lactation periods, and the results are shown in Figure 5. As seen in Figure 5A, the yak milk powder sample points from different groups formed three distinct clusters. Specifically, the YCSP sample points were in the third quadrant, the YEMP sample points were in the fourth quadrant, and the YMMP sample points were concentrated at the intersection of the first and second quadrants. Additionally, the calculated model parameters in the OPLS-DA model showed that the R2 value was 0.996, and the prediction ability parameter (Q2) was 0.906. This indicated that the prediction ability of the OPLS-DA model was highly reliable [37].

Although the OPLS-DA model was able to distinguish the differences in volatile compounds among the yak milk powder samples, we could not assess the robustness of the model, so a permutation test was still needed to verify whether the model was overfitted [37,38]. The permutation test is a method that involves random rearrangement of the sample data followed by statistical inference, which can increase the sample size in the model, typically set at *n* = 200 [39]. The permutation test results are shown in Figure 5B, where the horizontal axis represents the retention degree of the samples and the vertical axis represents the values of R2/Q2. After 200 cross-validations, the regression line of Q2 crossed the horizontal axis, and all Q2 and R2 values in the permutation test were consistently lower than the original Q2 and R2 values. Additionally, the slopes of the regression lines for R2 and Q2 were both greater than 1, and the intercept of the Q2 regression line was negative (−0.4065). These results indicated that the OPLS-DA model had good fit and strong predictive ability [36,39].

### 3.5. Analysis of Characteristic Volatile Compounds

**Figure 6:**
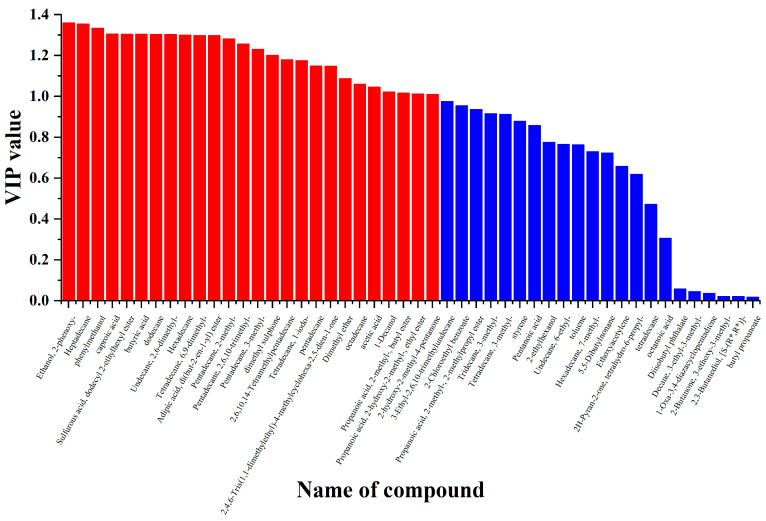
Variable importance in projection (VIP) results for 18 volatile compounds. Red bars highlight volatile compounds with VIP > 1.

In the OPLS-DA model analysis, we obtained the variable importance in projection (VIP) scores for each volatile compound, as shown in Figure 6. From the figure, it can be observed that 26 volatile compounds have VIP values greater than 1 (indicated by the red bars in the Figure 6).

VIP provides a significant reference value for identifying characteristic volatile compounds in samples. Generally, compounds with VIP > 1 are considered characteristic volatile compounds [40,41]. We selected 21 volatile compounds with VIP > 1 and *p* < 0.05, as shown in Table 2. These compounds include Pentadecane, 2-methyl-; Tetradecane, 6,9-dimethyl-; Pentadecane, 2,6,10-trimethyl-; 1-Decanol; Heptadecane; Hexadecane; Ethanol, 2-phenoxy-; Phenylmethanol; Caproic acid; Sulfurous acid, dodecyl 2-ethylhexyl ester; Butyric acid; Dodecane; Undecane, 2,6-dimethyl-; Adipic acid, di(but-2-en-1-yl) ester; Pentadecane, 3-methyl-; Dimethyl sulphone; Pentadecane; Dimethyl ether; Acetic acid; Propanoic acid; 2-Hydroxy-2-methyl-, ethyl ester; and 2-Hydroxy-2-methyl-4-pentanone.

**Figure 7:**
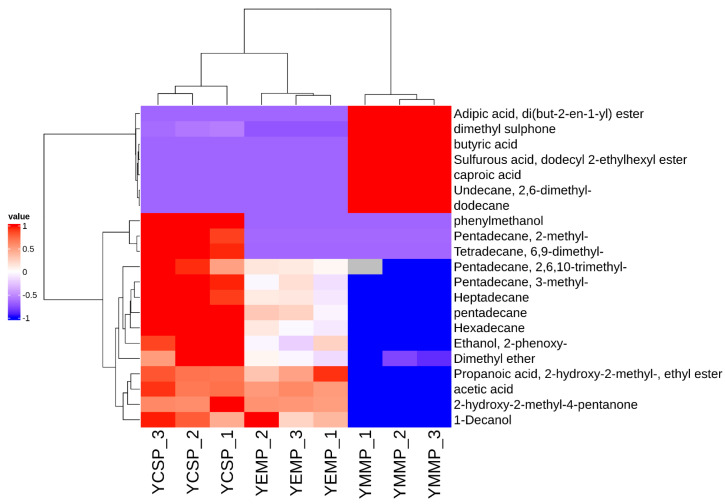
Hierarchical clustering heatmap analysis of identified volatile compounds in yak milk samples from different lactation periods. The vertical axis represents the sample numbers from different lactation periods, and the horizontal axis represents the names of the detected volatile compounds. Blue blocks indicate relatively low content, while red blocks indicate relatively high content.

We further performed hierarchical clustering analysis on the 21 characteristic volatile compounds, as shown in Figure 7. The results indicate significant differences in the characteristic volatile compounds among the YCSP, YMMP, and YEMP samples, with noticeable variations in the content of these compounds across different yak milk powder samples.

The results of the analysis of characteristic volatile compounds indicate significant differences among the YCSP, YMMP, and YEMP samples. Previous studies on flavor compounds in yak milk and yak milk powder have found that factors such as the origin of the milk [8], fermentation strains [2], sterilization methods [28], drying methods [27], and storage conditions [13] can all affect the types and concentrations of volatile compounds in yak milk. In this study, we controlled for these factors by using samples collected from yak milk powder at different lactation periods to specifically investigate the differences in volatile compounds.

Compared to volatile compounds in other milk powders, we found that yak milk powder has unique volatile compound characteristics, such as fatty acids and fatty acid esters, which may be related to its growing environment and feeding practices [42]. Although HS-SPME-GC-MS offers high sensitivity, it also has some limitations, such as potential interference from other components in complex samples and lower extraction efficiency for certain compounds. To improve detection accuracy, future studies could combine other analytical methods, such as liquid chromatography–tandem mass spectrometry (LC-MS/MS) [43]. The results of this study can help dairy manufacturers optimize their processes, especially in enhancing the flavor of yak milk, by selecting specific processing techniques or additives to retain key volatile compounds, ensuring consistency in flavor and quality.

In this study, the types and concentrations of volatile compounds in different yak milk powder samples determined their flavor and aroma. Fatty acid esters typically have sweet and fruity aromas, which are highly appreciated in dairy products [16]. The higher concentration of these compounds in YMMP may explain its richer, more complex flavor. Understanding how these compounds affect flavor will help improve the production processes and quality control of yak milk products. Additionally, this study did not focus on the significant impact of climate and feeding conditions on the composition of volatile compounds in yak milk powder. As this factor is related to the formation of flavor compounds in yak milk, future research should focus on how environmental factors influence the volatile components in yak milk.

It must be emphasized that the results of this study have important practical value for the dairy industry, particularly to produce high-quality yak milk powder. By understanding the volatile compound composition in yak milk at different lactation stages, producers can optimize processing conditions to enhance flavor while maintaining nutritional integrity. Moreover, the volatile biomarkers identified in this study can be used for quality control, ensuring consistency in flavor and aroma in commercial production. Additionally, by identifying the characteristic aromatic volatile compounds in yak milk powder, producers can adjust their manufacturing processes to create products that are more appealing to consumers. This is particularly important as yak milk enters the market as a new product, as understanding consumer flavor preferences can guide the production of products with broad market acceptance.

## 4. Conclusions

This study utilized headspace solid-phase microextraction and gas chromatography–mass spectrometry to identify and compare the volatile compounds in freeze-dried yak milk powder samples from different lactation periods. A total of 48 volatile compounds were identified, with significant differences in both the types and concentrations of these compounds, revealing distinctive characteristics of yak milk powder from different lactation stages. These findings provide valuable insights for flavor research and determining the optimal harvesting period for yak milk powder. Ester compounds were found to be more prevalent in mature yak milk powder, contributing to a more pleasant flavor. Based on these results, volatile compound analysis can be used to improve flavor and quality by optimizing the harvesting period and processing techniques, thereby guiding the development of new dairy products. Furthermore, this approach can help local producers enhance product consistency and add value. Future studies should explore the impact of feeding practices and climate on the volatile compound profile, which would support producers in optimizing their production strategies. Integrating volatile compound analysis into quality control processes can ensure high product quality and enhance market competitiveness.

## Figures and Tables

**Table 1:** Information on compounds identified in YCSP, YMMP, and YEMP samples.

Number	Retention Time (min)	Volatile Compounds	Formula	CAS ID	Yak Colostrum Powder	Yak Mature Milk Powder	Yak Ending Milk Powder
1	4.267	Dimethyl ether	C_2_H_6_O	115-10-6	12,703 ± 1538 a	3614 ± 259 b	15,599 ± 334 a
2	6.307	toluene	C_7_H_8_	108-88-3	830 ± 182 b	ND	1610 ± 34 a
3	9.419	butyl propanoate	C_7_H_14_O_2_	590-01-2	ND	ND	91.2 ± 14.5 a
4	10.962	dodecane	C_12_H_26_	112-40-3	ND	159 ± 10 a	ND
5	11.645	Propanoic acid, 2-hydroxy-2-methyl-, ethyl ester	C_6_H_12_O_3_	80-55-7	227 ± 61 b	ND	321 ± 56 a
6	12.803	styrene	C_8_H_8_	100-42-5	168 ± 38 b	ND	283 ± 8 a
7	13.565	2-Butanone, 3-ethoxy-3-methyl-	C_7_H_14_O_2_	36687-99-7	ND	ND	344 ± 11 a
8	16.279	2-hydroxy-2-methyl-4-pentanone	C_6_H_12_O_2_	123-42-2	156 ± 27 a	12.3 ± 1.3 b	220 ± 4 a
9	16.662	tetradecane	C_14_H_30_	629-59-4	162 ± 55 b	127 ± 6 c	221 ± 21 a
10	17.324	Propanoic acid, 2-methyl-, 2-methylpropyl ester	C_8_H_16_O_2_	97-85-8	11.1 ± 0.7 a	ND	ND
11	17.344	Propanoic acid, 2-methyl-, butyl ester	C_8_H_16_O_2_	97-87-0	13.9 ± 3.1 a	ND	ND
12	17.811	3-Ethyl-2,6,10-trimethylundecane	C_16_H_34_	1000432-25-9	216 ± 53 a	50.0 ± 3.3 b	299 ± 3 a
13	18.165	Tridecane, 3-methyl-	C_14_H_30_	6418-41-3	62.9 ± 15.0 a	12.1 ± 3.1 b	92.2 ± 22.3 a
14	18.387	Tetradecane, 3-methyl-	C_15_H_32_	18435-22-8	107 ± 28 b	21.7 ± 2.0 c	154 ± 21 a
15	18.407	5,5-Dibutylnonane	C_17_H_36_	6008-17-9	94.2 ± 16.6 a	23.0 ± 1.3 b	ND
16	18.83	acetic acid	C_2_H_4_O_2_	64-19-7	133 ± 39 b	ND	182 ± 8 a
17	19.281	pentadecane	C_15_H_32_	629-62-9	2330 ± 513 a	416 ± 38 b	2715 ± 173 a
18	19.446	2-ethylhexanol	C_8_H_18_O	104-76-7	99.1 ± 34.4 a	61.1 ± 7.2 b	92.6 ± 5.2 a
19	20.103	Decane, 3-ethyl-3-methyl-	C_13_H_28_	17312-66-2	ND	ND	67.0 ± 25.6 a
20	20.284	Tetradecane, 6,9-dimethyl-	C_16_H_34_	55045-13-1	326 ± 119 a	ND	ND
21	20.659	Pentadecane, 2-methyl-	C_16_H_34_	1560-93-6	231 ± 96 a	ND	ND
22	20.896	Pentadecane, 3-methyl-	C_16_H_34_	2882-96-4	201 ± 46 a	35.4 ± 0.7 b	213 ± 17 a
23	21.709	Hexadecane	C_16_H_34_	544-76-3	1841 ± 518 a	220 ± 28 b	1766 ± 80 a
24	21.975	2,3-Butanediol, [S-(R*,R*)]-	C_4_H_10_O_2_	19132-06-0	ND	ND	432 ± 17 a
25	22.243	Sulfurous acid, dodecyl 2-ethylhexyl ester	C_20_H_42_O_3_S	1000309-19-5	ND	36.5 ± 3.1 a	ND
26	22.249	Undecane, 2,6-dimethyl-	C_13_H_28_	17301-23-4	ND	35.2 ± 2.6 a	ND
27	22.278	Pentadecane, 2,6,10-trimethyl-	C_18_H_38_	3892-00-0	327 ± 123 a	ND	329 ± 11 a
28	22.518	Undecane, 6-ethyl-	C_13_H_28_	17312-60-6	88.9 ± 23.9 a	ND	96.3 ± 23.7 a
29	22.543	Hexadecane, 7-methyl-	C_17_H_36_	26730-20-1	103 ± 9 a	ND	123 ± 26 a
30	22.976	Tetradecane, 1-iodo-	C_14_H_29_I	19218-94-1	47.0 ± 10.6 a	ND	54.8 ± 14.8 a
31	22.997	butyric acid	C_4_H_8_O_2_	107-92-6	ND	302 ± 23 a	ND
32	23.407	2,6,10,14-Tetramethylpentadecane	C_19_H_40_	1921-70-6	166 ± 68 a	ND	186 ± 35 a
33	23.939	Heptadecane	C_17_H_36_	629-78-7	392 ± 107 a	23.8 ± 0.6 b	343 ± 32 a
34	26.095	octadecane	C_18_H_38_	593-45-3	44.3 ± 14.8 a	ND	ND
35	26.704	Ethoxyacetylene	C_4_H_6_O	927-80-0	ND	13.7 ± 0.7 b	33.4 ± 3.0 a
36	26.712	1-Oxa-3,4-diazacyclopentadiene	C_2_H_2_N_2_O	288-99-3	9.29 ± 4.52 b	13.8 ± 1.2 b	34.5 ± 4.8 a
37	27.682	Pentanoic acid	C_5_H_10_O_2_	109-52-4	53.0 ± 5.7 b	240 ± 13 a	69.3 ± 2.0 b
38	27.683	caproic acid	C_6_H_12_O_2_	142-62-1	ND	234 ± 13 a	ND
39	28.331	phenylmethanol	C_7_H_8_O	100-51-6	40.2 ± 8.4 a	ND	ND
40	28.705	2,4,6-Tris(1,1-dimethylethyl)-4-methylcyclohexa-2,5-dien-1-one	C_19_H_32_O	19687-22-0	46.2 ± 11.1 a	ND	58.8 ± 3.3 a
41	29.134	dimethyl sulphone	C_2_H_6_O_2_S	67-71-0	35.5 ± 3.7 b	271 ± 22 a	ND
42	29.899	1-Decanol	C_10_H_22_O	112-30-1	22.5 ± 7.9 a	ND	30.9 ± 6.7 a
43	31.863	octanoic acid	C_8_H_16_O_2_	124-07-2	67.4 ± 2.2 a	29.2 ± 8.5 b	ND
44	33.497	Ethanol, 2-phenoxy-	C_8_H_10_O_2_	122-99-6	1222 ± 167 a	102 ± 11 b	1117 ± 155 a
45	34.441	2H-Pyran-2-one, tetrahydro-6-propyl-	C_8_H_14_O_2_	698-76-0	13.0 ± 1.4 a	15.9 ± 5.0 a	ND
46	35.313	Adipic acid, di(but-2-en-1-yl) ester	C_14_H_22_O_4_	1000324-71-1	ND	14.6 ± 2.1 a	ND
47	38.476	2-Chloroethyl benzoate	C_9_H_9_ClO_2_	939-55-9	17.2 ± 8.4 b	34.5 ± 0.5 a	ND
48	40.075	Diisobutyl phthalate	C_16_H_22_O_4_	84-69-5	54.9 ± 10.7 b	50.9 ± 1.9 b	106 ± 21 a

ND = not detected. Concentrations are expressed in μg/kg. The values labeled with different lowercase letters indicate significant differences (*p* < 0.05).

**Table 2:** Information on characteristic volatile compounds in YCSP, YMMP, and YEMP samples (VIP > 1 and *p* < 0.05).

Number	Retention Time (min)	Volatile Compounds	CAS ID	Yak Colostrum Powder	Yak Mature Milk Powder	Yak Ending Milk Powder	*p* Value	VIP Value
1	20.659	Pentadecane, 2-methyl-	1560-93-6	231 ± 96 a	ND	ND	0.003	1.281
2	20.284	Tetradecane, 6,9-dimethyl-	55045-13-1	326 ± 119 a	ND	ND	0.002	1.298
3	22.278	Pentadecane, 2,6,10-trimethyl-	3892-00-0	327 ± 123 a	ND	329 ± 11 a	0.002	1.256
4	29.899	1-Decanol	112-30-1	22.5 ± 7.9 a	ND	30.9 ± 6.7 a	0.002	1.021
5	23.939	Heptadecane	629-78-7	392 ± 107 a	23.8 ± 0.6 b	343 ± 32 a	0.001	1.354
6	21.709	Hexadecane	544-76-3	1841 ± 518 a	220 ± 28 a	1766 ± 80 a	0.001	1.3
7	33.497	Ethanol, 2-phenoxy-	122-99-6	1222 ± 167 a	102 ± 11 b	1117 ± 155 a	0	1.36
8	28.331	phenylmethanol	100-51-6	40.2 ± 8.4 a	ND	ND	0	1.333
9	27.683	caproic acid	142-62-1	ND	234 ± 13 a	ND	0	1.305
10	22.243	Sulfurous acid, dodecyl 2-ethylhexyl ester	1000309-19-5	ND	36.5 ± 3.1 a	ND	0	1.304
11	22.997	butyric acid	107-92-6	ND	302 ± 23 a	ND	0	1.304
12	10.962	dodecane	112-40-3	ND	159 ± 10 a	ND	0	1.303
13	22.249	Undecane, 2,6-dimethyl-	17301-23-4	ND	35.2 ± 2.6 a	ND	0	1.303
14	35.313	Adipic acid, di(but-2-en-1-yl) ester	1000324-71-1	ND	14.6 ± 2.1 a	ND	0	1.298
15	20.896	Pentadecane, 3-methyl-	2882-96-4	201 ± 46 a	35.4 ± 0.7 b	213 ± 17 a	0	1.23
16	29.134	dimethyl sulphone	67-71-0	35.5 ± 3.7 b	271 ± 22 a	ND	0	1.201
17	19.281	pentadecane	629-62-9	2330 ± 513 a	416 ± 38 b	2715 ± 173 a	0	1.149
18	4.267	Dimethyl ether	115-10-6	12,703 ± 1538 a	3614 ± 259 b	15,599 ± 334 a	0	1.087
19	18.83	acetic acid	64-19-7	133 ± 39 b	ND	182 ± 8 a	0	1.045
20	11.645	Propanoic acid, 2-hydroxy-2-methyl-, ethyl ester	80-55-7	227 ± 61 b	ND	321 ± 56 a	0	1.012
21	16.279	2-hydroxy-2-methyl-4-pentanone	123-42-2	156 ± 27 a	12.3 ± 1.3 b	220 ± 4 a	0	1.01

ND = not detected. The values labeled with different lowercase letters indicate significant differences (*p* < 0.05).

## Data Availability

The original contributions presented in the study are included in the article, further inquiries can be directed to the corresponding author.
